# Bioconversion of Poultry Litter into Insect Meal and Organic Frasstilizer Using Black Soldier Fly Larvae as a Circular Economy Model for the Poultry Industry: A Review

**DOI:** 10.3390/insects16010012

**Published:** 2024-12-27

**Authors:** Anand Raj Kumar Kullan, Arumuganainar Suresh, Hong Lim Choi, Elke Gabriel Neumann, Fatima Hassan

**Affiliations:** 1 Department of Integrative Agriculture, United Arab Emirates University, Al Ain, Abu Dhabi P.O. Box 15551, United Arab Emirates; elke_neumann@uaeu.ac.ae (E.G.N.); fatima.hassan@uaeu.ac.ae (F.H.); 2 Resourcification Research Center for Crop-Animal Farming, Seoul 151-742, Republic of Korea; suresharumuganainar@gmail.com (A.S.); ulsoo8@snu.ac.kr (H.L.C.); 3 Department of Agricultural Biotechnology, Research Institute for Agriculture and Life Sciences, Seoul National University, Seoul 151-742, Republic of Korea

**Keywords:** bioconversion, black soldier fly larvae, poultry litter, entomoremediation, insect meal, frasstilizer

## Abstract

Poultry litter waste management poses a significant global challenge, attributed to its characteristics (odorous, organic, pathogenic, attracting flies). Conventional approaches to managing poultry litter involve composting, biogas generation, or direct field application. Recently, there has been a surge of interest in a novel technology that involves the bioconversion of organic waste utilizing insects (known as entomoremediation), particularly focusing on black soldier fly larvae (BSFL), and has demonstrated successful transformation of various organic waste materials into insect meal and frass (referred to as organic frasstilizer). Black soldier flies have the capacity to consume any organic waste material (ranging from livestock litter, food scraps, fruit and vegetable residues, sewage, sludge, municipal solid waste, carcasses, and defatted seed meal) and convert it into valuable BSFL insect meal (suitable for animal feed) and frass (serving as an organic fertilizer). The bioconversion of poultry litter by black soldier flies offers numerous advantages over traditional methods, notably in terms of reduced land and water requirements, lower emissions, cost-effectiveness, swift processing, and the production of both animal feeds and organic fertilizers. This review focuses on the existing knowledge of BSFL, their potential in bioconverting poultry litter into BSFL meal and frass, and the utilization of BSFL in poultry nutrition, emphasizing the necessity for further innovation to enhance this sustainable circular economy approach.

## Simple Summary

Poultry litter waste management is a global challenge due to its odorous, organic, and pathogenic nature. Traditional management methods include composting, biogas production, and direct field application, all of which have certain drawbacks. A new technology called entomoremediation uses insects, particularly black soldier fly larvae (BSFL), for waste management. BSFL can consume various organic wastes, from livestock litter to food waste and carcasses. The process converts organic waste (poultry litter) into two valuable products: insect meal (for animal feed) and frass (organic fertilizer). This bioconversion method offers significant advantages over conventional approaches, such as reduced land and water usage, lower emissions, cost-effectiveness, faster processing, an a sustainable approach based on circular economy principles. Further research and innovation are needed to enhance this promising waste management solution.

## 1. Introduction

A 73% surge in the consumption of animal products is anticipated by the year 2050, according to the Food and Agriculture Organization [1]. Within the category of meats, poultry consumption is projected to exceed that of both beef and pork [2]. The rising expenses associated with chicken feed, which accounts for 60–70% of the total production costs, raise concerns regarding the sustainability of chicken farms and the related environmental implications [3]. Chicken feed typically consist of plant-based components such as soybean, corn, wheat, and animal-based ingredients like fish, meat, and bone meal, including proteins, oils, and their combinations [4]. Furthermore, feed costs are escalating rapidly, having doubled within a short timeframe. Moreover, the availability of feed is progressively declining, posing challenges for producers [5]. Increased production of plant- and fish-based meals could potentially lead to elevated deforestation, diminished soil fertility, and overfishing contributing to species extinction, thereby exerting significant pressure on our finite natural resources amidst ongoing climate changes and the competition between food, feed, and fuel resources [6]. Consequently, the development of a sustainable and cost-effective alternative feed option would be advantageous for chicken producers seeking profitability [7].

The poultry sector is generating a substantial quantity of poultry litter (PL), an organic waste (OW), utilized primarily as fertilizers, some for energy generation, and repurposed as animal feed post-treatment in the context of a circular economic model [8,9]. The mismanagement of OW from poultry farming has adverse effects on the atmosphere (via odor emissions), water bodies (nutrients, OM, pathogens), and soil (pathogens), serving as contaminants [10]. Thus, the question arises: can OW from the poultry sector be converted into a source of protein? Indeed, saprophagous insects have the capacity to consume this waste and transform it into protein, fat (used as animal feed), and residual matter (organic fertilizer), and this process (entomoremediation, using insects for pollutant remediation) has gained significant attention over the last decade [11,12]. Insects represent the most abundant and diverse category of creatures on the planet [13] and concurrently are an extensive yet underexploited valuable asset [14] among these small non-domesticated creatures known as ‘minilivestock’ [15]. In few parts of Asia, insect protein can be directly consumed as food, although insects are considered odd table menu in various parts of the world. Therefore, by utilizing these insects as feed for animals (such as chickens, fish, and pigs), they can be converted into meat protein [16]. Additionally, insect meal has the potential to be more cost-effective and nutritionally superior compared to vegetable meal due to its abundance of essential amino acids and its eco-friendliness and sustainability [6].

The black soldier fly (BSFL) is highly regarded among insect feed options due to its ease of rearing, ability to consume various organic materials, superior quality, efficiency, nutritional value, eco-friendliness, rapid growth, and minimal resource requirements [6,17,18,19]. In terms of processing, quality, and environmental impact, the BSF is superior to the other insects, such as the house fly, which turn trash into insect meal (Table 1). The capacity of BSFL to efficiently convert organic waste into larval biomass at a rate of approximately 15% has positioned them as a key species for large-scale production [20]. This biomass could serve as an alternative food and feed source if deemed safe and permissible under regulations [21]. The approval of BSFL for animal feed by the US and EU, driven by its nutrient richness and immunostimulant properties, further underscores its value [22,23]. Studies indicate that integrating BSFL meal into animal feed enhances meat quality, animal welfare, health, and feed conversion ratios [24]. Additionally, the byproduct known as frass, containing beneficial microbes and a wealth of nutrients and minerals, can be added as soil amendments [25,26].

**Figure 1:**
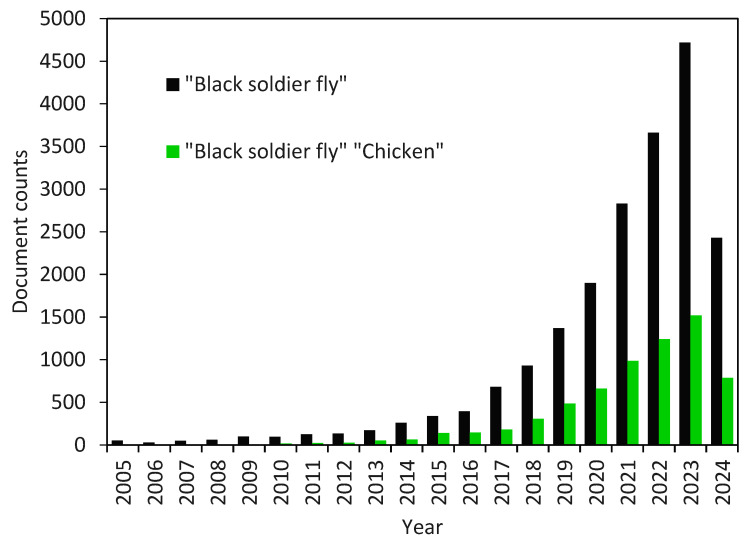
Number of document counts on “Black soldier fly” and “Black soldier fly” “Chicken” for the period of 2005–2025 from the Google Scholar database using custom range, accessed 3 July 2024 (https://scholar.google.com/).

The production of black soldier fly larvae meal (BSFLM) can be derived from various types of organic waste, although it necessitates a foundational understanding of the insect [3]. Recent trends show a growing interest in BSFL research and the cultivation of their larvae through the utilization of organic waste (Figure 1). This review article delves the biological aspects of the BSF, the rearing process utilizing PL as a substrate, the potential yield capacity, the bioconversion rate (BCR), and waste reduction efficiency. Nonetheless, there is a requirement for enhancements in techniques to facilitate production, enhance BCR, and increase yield [27], as well as to identify gaps in knowledge within this field. The insights provided in this review can offer valuable assistance to the poultry sector in promoting sustainable development by effectively managing their organic waste to produce insect meal (IM), thereby contributing towards the realization of a circular economy.

**Table 1:** Comparison of black soldier fly vs. house fly in waste bioconversion.

	Black Soldier Fly	House Fly	Reference
Waste as substrate	Diverse organic waste	Few organic waste	[28]
Gut microbiota	Specialized	Not specialized	
Waste processing rate	High (15–25 kg waste/m^2^/day)	Low (3–5 kg waste/m^2^/day)	[29]
Waste reduction (%)	High (55–75%)	Low (30–50%)	
Disease transmission	No (adult flies do not feed)	Yes (known as vector for disease spread)	[29]
Attract human settlements	No	Yes	[17]
Odor problem	Less	More	
Quality of endproducts (meal and frass)	High	Low	
Ability to kill pathogens	Yes	No	[30,31]

## 2. BSF Lifecycle, Their Biology, and Rearing Knowledge

Some crucial biological and practical knowledge about the BSF must be acquired before engaging in rearing for its commercial significance. The black soldier fly (BSF, *Hermetiaillucens*) is indigenous to the Americas and is prevalent across tropical and temperate zones [32]. The BSF has been introduced and established in Australia, India, Africa, and Europe [33,34], demonstrating its adaptability to a wide array of environmental conditions such as light, temperature, and relative humidity (RH).

The biology of BSFL has been extensively studied over the last two decades, particularly in relation to its applications in waste management and protein generation. While only a few industries produced insect protein a decade ago, today, numerous organizations are involved in research activities (refer to Figure 1) and investments related to BSFL meal. The global market for BSFL is projected to reach 3.4 million tons by 2030 [35]. Successful cultivation of BSFL necessitates a profound understanding of insect biology and substantial investments to ensure both quality and quantity in production. Commercial-scale production of BSFL insect meal involves various stages such as fly growth and egg production (insectarium), larva production (larvarium), and larva processing (drying, grinding, pelleting). Practical knowledge is essential during rearing in both small and large operations, including the addition of moisture-retaining materials (e.g., cocopeat or sawdust) on top of the organic waste to prevent water loss and enhance air circulation, the inoculation of eggs or larvae of the same age, and the harvesting of larvae upon the first appearance of prepupae to achieve optimal protein yield and digestibility [36]. Post-harvest, it is crucial to empty the larval gut after a fasting period of approximately 24–48 h to obtain high-quality insect meal [37]. Additionally, 10–20% of the larvae should be preserved to sustain the colony through pupation, leading to the emergence of adult flies.

**Figure 2:**
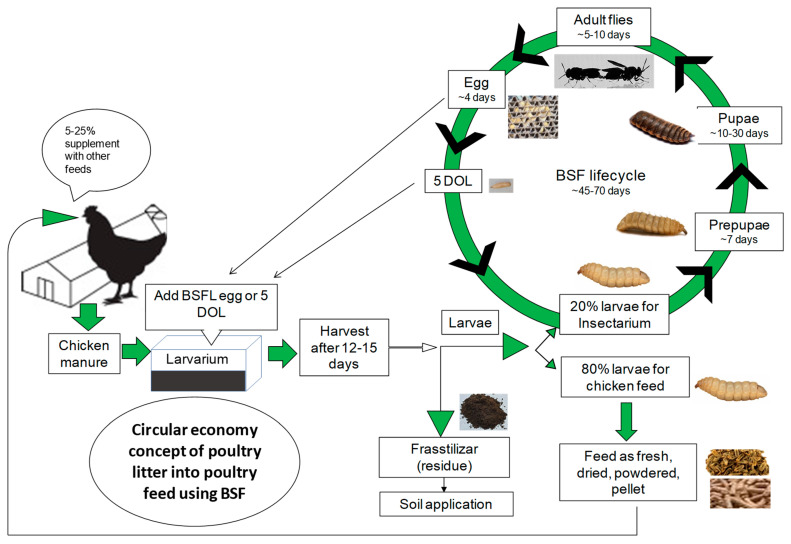
The circular economy concept of bioconverting poultry litter into black soldier fly larvae meal, frasstilizer, and lifecycle of black soldier fly.

The BSF is a tropical insect endemic to certain regions, resembling a wasp, which serves as a defense mechanism against predators. This species is considered a non-synanthrope, is neither a pest- nor disease-carrying vector, and neither stings nor bites [16]. Adult BSFs do not consume solid food; instead, they utilize their stored fat reserves for energy expenditure during mating and flying. Conversely, the larvae of the BSF (saprophagous) exhibit a voracious appetite, consuming up to twice their body weight daily and consuming a wide-ranging organic waste (polyphagous) as their diet [38]. The digestive extracts of BSFL larvae display significant enzymatic activities, including amylase, lipase, and protease functions. Research indicates that a single BSFL spends 15 min to feeding per hour, resting for the remaining 45 min near food sources, thereby allowing other larvae accessing the nourishment [39]. Throughout their larval stage lasting approximately three weeks, black soldier fly larvae efficiently consume all the food required for their subsequent life stages (Figure 2). The survival and growth of BSFL are contingent upon various factors such as temperature, relative humidity, and the characteristics of the substrate, including food type, composition, and availability [19,40]. The lifecycle of the BSF, as illustrated in Figure 2, typically concludes within a span of approximately 45 to 70 days. Adult BSFs have a brief lifespan of around 5 to 10 days; however, certain studies have shown an extension of their longevity through provisions of water, milk, and honey syrup [41,42,43]. During the brief lifespan of the adult flies, both male and female counterparts engage in mating activities and deposit eggs in crevices and dry cracks near decaying organic waste [44]. An estimated average of around 500 eggs are laid per oviposition, with each cluster of eggs weighing approximately 15 mg and individual eggs weighing about 25 µg. It is observed that females usually mate once and perish post-oviposition [41]. The eggs are anticipated to hatch within a span of 3–5 days, leading to the emergence of white-colored day-old larvae (DOL) measuring approximately 1 mm in length. These larvae voraciously feed to attain the black color prepupal stage (non-eating migratory phase) within 15–20 days, sized 1.2 cm and weighing 150 mg. Subsequently, they move from a moist environment to a dry location employing a hook mechanism formed by their converted mouth, ultimately transforming into pupae (non-moving stage) over a period of about 7 days. Sheppard et al. [32] recommended a 40-degree slope for efficient self-harvesting of prepupae. The pupal stage, a motionless phase spanning approximately 10–30 days, facilitates metamorphosis, culminating in the emergence of the adult fly. Tomberlin and Sheppard [41] highlighted that mating takes place two days post-eclosion, followed by oviposition after an additional two days post-mating. Recent research by Julita et al. [45] delved into the mating behavior of the BSF, unveiling intriguing details of a 2–8 day mating period occurring at temperatures of 27–29 °C between 9:00 am and 14:00 pm. The peak mating activity was noted among 2–3-day-old flies around 11:00–12:00 due to optimal sunlight exposure aiding in female detection, with an average coupling duration of 36 min. Oviposition commences from the fourth day post emergence (two days after mating), extending up to the tenth day, peaking from 13:00 to 14:00 pm and lasting until 17:00 pm, with an average egg count ranging between 569 and 650 per female, each measuring 1 mm in length. The mean lifespan of an adult fly was recorded at 12–14 days when provided solely with water, while the average developmental duration from egg to adulthood was approximately 55 days.

Research has indicated that the reproductive success and lek behavior of the BSF can be impacted by various abiotic and biotic factors, including light intensity [46,47,48], relative humidity [49], temperature [50], the size and spatial dimensions of the cage [46,51], and the density of adult flies. Hoc et al. [52] have proposed that a density of 0.013 flies/cm^3^ (equivalent to 13,000 flies/m^3^) could be considered optimal for egg production. Adult flies engage in mating activities primarily during daylight hours, requiring light with 60% relative humidity and a temperature of 27 °C as the ideal conditions [51,53]. Sunlight is the best source for the light; however, artificial lights can be used. The intensity of artificial light, at 200 μmol m^–2^ s^–1^ (10,800 lux), played a major role in finding females [54]. Likewise, BSF mating success can be dramatically increased by exposure to rich-wavelength lights near 440 and/or 540 nm and having irradiance equivalent to the intensity of full sunlight [46]. In the insectarium, water needs to be provided in a socked cotton cloth, as adult flies drink by sucking, and milk or honey syrup can be added for more longevity [41,42,43]. Females do not lay eggs on the decaying waste; therefore, eggies (material for laying eggs, such as corrugated cardboard or layered wood or plastic) must be provided near the organic waste [29,49] which provides protection from the predators and immediate feed to the larvae after hatching.

Muyou [55] provided information regarding the preservation of BSF eggs in cold temperatures between 0 and 10 °C and at a relative humidity (RH) of 70–80% for 25 days. During this storage, the hatchability of the eggs reached a peak of 43%, whereas the control group (without cold treatment within the normal hatching period of 4–7 days) exhibited a hatchability of 70%. Extending the preservation duration of BSF eggs, while maintaining high hatchability rates, could enhance breeding efficiency with reduced labor requirements. Further research is necessary to optimize the conditions for continuous egg storage in the system.

Larvarium is a development of larvae originating from the egg and transitioning into the pupal stage; during these phases, the larvae exhibit photophobic behavior while necessitating oxygen. Consequently, to ensure optimal larvae growth, it is recommended to maintain a waste depth of less than 7 cm, thereby averting oxygen deprivation and preventing overheating, whether in a plastic tray (measuring 60 cm × 40 cm × 12 cm) or a concrete pit (1 m × 1 m × 12 cm). It is essential to create a light-restricted environment by utilizing a breathable cloth covering to facilitate the complete consumption of organic waste. In general, organic waste tends to attract houseflies and dominates the surroundings; however, the existence of BSFs and BSFL in the organic matter acts as a deterrent to *Musca domestica* L., courtesy of certain repellent chemicals emitted by BSFL, effectively repelling houseflies and other similar insects [56,57]. Furthermore, the inhibition or reduction of pathogenic bacteria in the organic waste can be achieved through the BSFL which posses antimicrobial peptides and a complex digestive enzyme complex [30,31,58], ultimately leading to a significant mitigation of odor-related issues.

According to Bosch et al. [36], prepupae have lower protein content than larvae, and there is a similar tendency in the in vitro digestibility data, with larvae showing higher levels of digestibility (84.3%) compared to prepupae (68.1%). This suggests that harvesting at the onset of the first prepupa would produce the best larvae production with the fewest prepupae and, consequently, the highest digestibility. Table 2 illustrates the nutritional components of BSFL (on a dry weight basis) cultivated in diverse organic waste substrates as indicated by various research studies. Evidently, there exists variation in the nutritional composition among different organic feeds. The moisture content of fresh larvae typically ranges from 20% to 44% [59,60,61] and is influenced by both the diet and the developmental stage of the larvae. The dry weight of BSFL generally comprises approximately 45% crude protein (CP) and 35% lipids and exhibits an amino acid profile akin to that of fishmeal, alongside fiber (5%), ash (10%), minerals (Fe, Zn) [62], and chitin [63]. The crude protein content tends to decline with larval age, with the highest proportion recorded in 5-day-old larvae (61%), while lower levels were observed in larvae aged 15 (44%) and 20 (42%) days [39]. Nonetheless, these nutritional attributes can be subject to variations based on the quantity, quality, and nature of the substrate [54,60,64,65,66], Nevertheless, BSFL has gained recognition and is being utilized as an alternative protein source in the feed for poultry, swine, fish, pets, and reptiles [67]. It represents a viable and economically beneficial substitute for fishmeal in animal feed additives, with an estimated market value of USD 330 per tonne of dry weight around USD 1500 per metric to in 2016 [68,69]. According to the most recent publication by Meticulous Research [30], it is anticipated that the global black soldier fly (BSF) market will experience a Compound Annual Growth Rate (CAGR) of 34.7% from the year 2020, reaching a value of USD 3.4 billion by the year 2030. BSFL are known to possess elevated levels of various minerals in comparison to other insect species [70], including manganese (Mn), iron (Fe), zinc (Zn), copper (Cu), phosphorus (P), and calcium (Ca), with the highest Ca/P ratio recorded at 8.4 [6]. The notable calcium content in BSFL can be attributed partially to the secretion of calcium carbonate (CaCO_3_) deposits by the epidermis of the BSFL, potentially justifying the high levels of calcium and ash content [71]. Additionally, lipids can be derived from the larvae for the production of biofuels [72], with lauric acid, saturated fatty acids, and EPA and DHA being prevalent [64]. The residue left behind post-larvae collection (known as frass) holds the potential to serve as an organic fertilizer (referred to as frasstilizer, this review) [25,26,73], thus opening up various alternative applications for the BSF.

Some fundamental equations need to be employed in the BSFL rearing system utilizing organic waste (OW), including waste reduction percentage (WR %), waste reduction index (WRI), larval survival rate in the organic waste (SR %), bioconversion rate (BCR %), growth rate (GR), feed conversion ratio (FCR), duration of development, and efficiency of frass production (EFP %).

(1) $$WR\left(\%\right)=\frac{Wi-Wf}{Wi}\times 100$$

where *Wi* is the initial weight of waste added, and *Wf* is the final weight of waste that remains.

(2) $$WRI=\frac{WR}{t}\times 100$$

where *t* is the total duration of feeding (days).

(3) $$SR\left(\%\right)=\frac{Ni-Nf}{Ni}\times 100$$

where *Ni* is the initial number of larvae added, and *Nf* is the number of larvae that remains after harvest.

(4) $$BCR\left(\%\right)=\frac{Weight\;of\;Larvae}{Weight\;oforganic\;waste\;added}\times 100$$

(5) $$GR=\frac{Weight\;of\;Larva}{t}$$

(6) $$FCR=\frac{Feed\;consumed\;\left(dry\;weight\right)}{Weight\;gained\;\left(live\;weight\right)}$$

(7) $$EFP=\frac{Wf}{Wi}\times 100$$

Larvae developmental time equals days between the inoculation of eggs or larvae and the first prepupae observed.

## 3. Bioconversion of PL into BSFL

The increase in poultry farms has resulted in a significant amount of litter production, and its inadequate management presents environmental risks due to the presence of organic matter, nutrients, pathogens, and malodorous compounds [10,87]. The PL contains over 50% of undigested feed, attributed to their shorter digestive system compared to other animals, resulting in a feed passage rate of less than 24 h. Adult chickens defecate every 15–25 min (~15 times/day) during their active phase, producing approximately 120 g–170 g per day [88,89]. The characteristics of poultry litter vary significantly across different locations (Table 3). Fresh PL comprises 55% moisture and 45% solids, with 87% of the solids being volatile, suitable for bioconversion into insect meal using BSFL [90]. BSFL demonstrate potential in efficiently transforming organic manure into high-quality insect meal rich in protein, fat, fiber, and minerals (Table 2). They also help in pathogen reduction, fly control, odor mitigation, and the production of organic fertilizer residues (frass, known as “frasstilizer”). Studies indicate that animal manure is a superior feed source for BSFL compared to vegetable waste, leading to improved survival rates, shorter developmental times, higher larval weights, and enhanced feed conversion ratios (FCRs) [3,20,31].

Significant investigations have utilized the bioconversion of poultry litter (PL) using BSFL into insect meal and assessed the yield, BCR, FCR, and WR through the natural process of entomoremediation (Table 4). Initially, Sheppard et al. [32] reported a bioconversion rate of 7.8% of PL into BSFL protein (42%) and fat (35%). This study was conducted directly within the chicken farm’s cage litter, resulting in an annual yield of 528 g of BSFL meal per chicken, estimating at 53 tons for every 100,000 chickens. It was also proposed that the litter of laying hens serves as an ideal substrate for raising BSFL without the need for specialized facilities or additional energy consumption and that expanding the operation scale led to enhanced bioconversion rates. Similar to the above studies, Miranda et al. [20] achieved a 45% protein content in BSFL from PL within a 12-day period, whereas a commercial feed (Gainesville diet/house fly diet) demonstrated 48% protein within 9 days. Furthermore, comparable waste reduction (46%), BCR (5.6%), and FCR (3.47) were observed when compared to the control feed, indicating the significant potential of BSFL in managing PL towards the concept of a circular economy.

Zhou et al. [105] discovered a significantly high WR of 48.4% (dry matter basis) when utilizing PL as raw material, whereas cow manure and pig manure showed 40% and 46% WR, respectively. In their investigation, the authors employed three distinct species of BSFL (from Texas, USA, and Guangzhou and Wuhan in China) along with three different types of organic waste (poultry, pig, and cow manure). The study concluded that PL was the most suitable organic waste for the cultivation of BSFs (Table 4). Oonincx et al. [61] processed oven-dried and ground PL to achieve uniformity, followed by the addition of water to reach a 66% moisture level. Subsequently, day-old larvae were introduced, resulting in low WR (37%), BCR (3.4%), and enhanced FCR (2.3). Surprisingly, the developmental duration was 144 days, contrasting with only 20 days for the control group fed chicken feed. The authors concluded that the oven-drying of the animal manure to feed BSFL could have led to a reduction in nutritional value due to the destruction of microorganisms and heat-labile vitamins. Therefore, it is highly advisable to choose fresh manure, as this may result in a considerably reduced development time and improved bioconversion efficiency

Rehman et al. [91] conducted a study on the simultaneous digestion of poultry litter (PL) with dairy manure (DM) utilizing BSFL and achieved optimal FCR of 6.63, BCR of 7.86%, and WR of 52.1% when the mixture contained 60% PL and 40% DM. In contrast, PL alone exhibited FCR, BCR, and WR values of 5.56, 9.88%, and 55%, respectively. The feeding ratio was maintained at 1 kg of waste per 1000 larvae until the first prepupae stage. Notably, the survival rate of larvae was significantly higher in PL (98.1%) compared to DM, and the developmental period was 4 days shorter in PL than in DM (22 days). Another investigation by Xiao et al. [92] revealed that the conversion of PL into BSFL biomass was 15.3%, surpassing that of pig and cow manure. Notably, the combination of PL with other manure sources did not improve the benefit–cost ratio; rather, it resulted in a considerable decline. Moreover, the incorporation of rice bran (15%) into PL resulted in an increased BCR of 20%. Miranda et al. [20] studied the industrial scale bioconversion of animal manure and found PL is better than PM and DM in terms of survivability of the larvae and larval weight gain and suggested for the large-scale production for the BSFL using animal manure. Recently, Wu et al. found similar findings that PL is a better substrate than PM in terms of BCR and WR, contradictory to Diola et al. [80] where PM is better than CM in terms of BCR and WR. This difference might be due the substrate conditions, sources, environmental factors, and the age of the nursery larvae used.

Nana et al. [102] discovered that batch feeding (administered all at the onset) exhibited a superior FCR of 7.1 compared to the FCR of 8.2 observed with continuous feeding (feeding once in 3 days for 15 days). Furthermore, the feeding amount of 160 mg per larva per day proved to be more effective in batch feeding compared to 100 mg and 220 mg PL/larvae. Diener et al. [59] determined that the optimal feeding ratio is 100 mg of waste per larva/day. Shumo et al. [79] fed PL, spent grain from breweries, and kitchen waste to BSFL. This study revealed distinct patterns of mineral accumulation, with PL yielding the highest turnover of minerals. It was also noted that the protein content of BSFL fed with PL surpassed that of commonly utilized livestock feeds like canola, cottonseed, and sunflower meal derived from plant sources. Similarly, the fat content of BSFL reared with PL exceeded that reported in full-fat soybean meal [106] and commercially available fishmeal [107].

Bortolini et al. [101] conducted an optimization study on the proportions of PL, water, and chabazite (a type of zeolite) to enhance the growth of BSFL. The study identified the optimal combination of 34.5% PL, 58.3% water, and 7.2% chabazite for maximizing BSFL production. Chabazite was found to be effective in mitigating odor issues by adsorbing compounds such as ammonia and H_2_S, thereby reducing unpleasant odors in the surroundings. While a maximum WR of 75% was achieved, the FCR was relatively high at 14.5, and the BCR was low at 5.2%.

### 3.1. BSFL and Associated Symbiotic Bacteria

The symbiotic relationship between black soldier fly larvae (BSFL) and the microorganisms found in their gastrointestinal tract and the surrounding environment is essential for nutritional metabolism, particularly in the processes of protein breakdown and assimilation. Mazza et al. [100] utilized BSFL in combination with their symbiotic bacteria to convert PL into insect meal. The study revealed that the incorporation of BSFL companion bacteria resulted in enhanced larval weight gain (28.6%), reduced waste (4.3%), improved bioconversion rates (1.89%), and increased nutrient accumulation (1.53%) compared to the control group which solely utilized BSFL. These findings underscore the importance of introducing BSF-associated bacterial strains (such as *Kocuria marina*, *Lysinibacillusboronitolerans*, *Proteus mirabilis*, and *Bacillus subtilis*) to facilitate nutrient accumulation in larvae and enhance PL management for BSFL producers. In a related study, Xiao et al. [104] conducted a large-scale experiment involving 1 ton of PL and 1 million BSFL (6 DOL) supplemented with 1 L of a bacterial solution (10^6^ CFU/mL) of *Bacillus subtilis* derived from the gut of BSFL. This intervention led to improved WR (40.5%) and BCR (11.5%) by 13.4% and 15.9%, respectively, compared to the control group (without bacteria) within a 13-day rearing period. Kooienga et al. [108] noted that the supplementation of *Arthrobacter*, a probiotic bacterium, resulted in a twofold increase in the growth rate of BSFL and a 25–30% improvement in waste conversion, while not affecting the microbial community. The probiotics enriched the functionalities related to protein digestion and absorption in the organic waste. Conversely, the probiotic *Bifidobacterium breve* had a contrasting effect, leading to a 50% decrease in larval weight gain and 20% lower bioconversion. Therefore, the selection of specific microbial strains for enhancing BSFL production needs further investigation. Tanga et al. [109] discovered that the composition of bacterial and fungal communities in the BSF larval gut exhibited significant variations among different substrates such as PL, kitchen waste, rabbit manure, and brewers’ spent grain. Despite this variability, a few microbial members remained consistently present across the substrates, indicating the existence of a potentially unique core gut microbiota in BSFL.

### 3.2. BSFL in Composting

Liu et al. [103] conducted a composting process on poultry litter (PL) utilizing BSFL at a specific ratio of 1.2:7 BSFL to PL, resulting in improved nutrient content and a 20% decrease in organic waste (OW). A comparison with traditional microbial composting revealed that the utilization of BSFL in composting was more effective as a fertilizer. The decline in organic matter (OM) can be primarily attributed to the respiration activities of microorganisms within the insects’ intestines, a phenomenon also documented by Rehman et al. [91]. The BSFL play a crucial role in the reduction in organic matter content. By engaging in muscular activities, the BSFL effectively degrade and homogenize substrates, thereby increasing surface area for microbial activities and facilitating OM reduction [110]. Furthermore, it was suggested that the compost produced from PL by BSFL (known as frass) would benefit from additional microbial composting to further enhance OM reduction, nutrient content, and germination index. Several research studies have highlighted that the application of BSFL frass can significantly improve soil fertility, plant growth, yield, and nutrient absorption [25,26]. Supporting the use of frass as soil amendments, Zhang et al. [111] discovered that composting PL with BSFL led to a notable decrease in microbial diversity while increasing the presence of organic matter-degrading microbes from the insects’ intestines. They recommended the adoption of BSFL composting for animal manure as a promising technology within the circular economy framework.

In general, PL contains pathogens such as *Salmonella* sp., *Eschericia* sp., *Campylobacter* sp., *Staphylococcus* sp., *Clostridium* sp., *Listeria* sp., *Actinobacillus* sp., and *Mycobacterium* sp. [112]; hence, generating BSFL with PL is a major worry for disease transmission. However, Awasthi et al. [31] discovered that composting PL with BSFL significantly reduced (90%) harmful microorganisms. Another study by Erickson et al. [30] found that BSFL rising in PL inactivated and reduced the *E. coli* and *Salmonella* strains, but there was no reduction in cow and hog manure. Even still, BSFL reduces (35%) the antibiotic resistance bacteria in the poultry litter and thus provides an effective measure for the reduction in antibiotic resistance in animal manure [113]. This research supports that the BSFL production utilizing PL is safe; has fewer pollutants, pathogens, and emissions; and is as profitable as quality insect meal. It was also advised that the BSFL production systems should be developed near the poultry farm in order to save money on transportation and increase sustainability.

Parodi et al. [114] provided initial evidence suggesting that BSFL exhibit a clear preference in their feeding behavior. The inquiry arises as to whether BSFL exhibit a preference for specific types of waste (such as organic waste) over others (like mass-rearing plant feed) when presented with a choice. Notably, it was observed that BSFL displayed a preference for malodorous organic waste of animal origin rather than the plant-based mass rearing feed, with this preference intensifying as the larvae aged. For example, when considering a sample of 500 larvae, 73% and 99% of them demonstrated a preference for organic waste at the ages of 9 and 16 days, respectively. The pronounced inclination towards animal manure may hold significant implications for the insect industry, the livestock sector (in terms of waste management and feed formulation), and other relevant stakeholders.

## 4. BSFL as Poultry Feed and Growth Performance

Chicken is the most economically viable meat for human consumption because of its high efficiency, with an FCR ranging from 1.5 to 1.8 [115]. Insect meal (FM) has become increasingly popular in the poultry market recently, surpassing plant- and fish-based meal [76]. Among insects, BSFL are a promising feed supplement for poultry [116]. BSF was highly recommended as animal feed, since it avoids human interaction and is generally not regarded as a pest or a carrier of disease. It also has a great deal of potential to provide a sustainable source of food for humans [117]. Furthermore, the approval granted in 2018 by the USFDA and the Association of American Feed Control Officials for the use of BSFL in chicken feed enhances its potential as a more sustainable protein source [86]. There are studies on the growth performance of BSFL as chicken feed available (Table 5). As a feed supplement for young chicks, broilers, layers, and other stages of hens, BSFL and prepupae raised on OW have been utilized with success [22,37,118].

### 4.1. BSFM in Broilers

The broilers that were provided with commercial diets, which include a partial substitution of BSFL for soymeal, soybean oil, and fishmeal (FM), resulted in improved production, reduced mortality, and good carcass traits [75,119,122,124]. BSFLM is a desirable element for use in the formulation of broiler feeds due to its high apparent metabolizable energy, richness in important amino acids and fatty acids, and apparent ileal digestibility [124,125]. Furthermore, in accordance with the broiler mineral needs Arango Gutiérrez [75] proposed that BSFLM has an adequate mineral content for the growth of poultry (Ca, Fe, Zn).

Specifically, Sumbule et al. [119] fed chicks and growers using varying amounts of BSFLM and FM and discovered that the birds who received a diet containing 25% BSFLM and 75% FM gained the most weight. The FCR in this feed fraction was 2.78 and 1.45 in the chick and grower stages, while FM alone demonstrated 3.17 and 1.64, respectively. Feed mix with higher BSFLM inclusion levels (>75%) showed positive cost–benefit ratios and returns on investment, which supports the poultry industry. According to Velten et al. [120], day-old chicks were fed partially defatted 50% BSFL with SBM and combined with amino acid supplements for a duration of 34 days. Overall, 50% BSFL substitution outperformed 100% SBM diets in terms of feed efficiency and growth response, achieving 1.26 and 1.72 FCR, respectively. At every stage of the bird’s development—starter, grower, and whole—the 50% BSFL supplements greatly improved FCR.

In a different trial, Dabbou et al. [80] fed day-old chicks with partially defatted BSFL with SBM at percentages of 10% and 15% for 35 days. Better growth and feed conversion ratios (FCRs) were demonstrated by the 10% BSFL substitution. The FCR was 1.16 for starters (1–10 days), 1.47 for growers, 1.83 for finishers, and 1.60 for all growth stages combined. Aging increased the daily weight gain, but the FCR was better in the beginning, and overall day gain was 62.5 g/day, which was consistent with the earlier study of 50% BSFL inclusion [120]. Nevertheless, there were no discernible effects of BSFL supplementation on the histological observations or hematochemical markers. Similarly, Popova et al. [126] used grower and finisher broiler chicken feed (14 to 35 days old) with partially defatted BSFL (5%) as well as full-fatted BSFL (5%). The broilers’ performance at 21 and 35 days was enhanced by adding 5% of partially defatted and full-fatted BSFL to their diet. In the groups that received the BSFL, there was a higher dressing percentage and carcass weight. The findings showed that the defatted and fattened BSFL had the ability to change the broiler carcass composition. Their inclusion in the diet of the birds tended to increase the percentage of the breast cuts, whereas the thigh muscles decreased significantly. In the study conducted by Schiavone et al. [122], soybean oil was substituted with 50% BSFL and 100% BSFL, and the broiler growth was examined (finisher 21–48 days). The authors concluded that the growth performance, carcass characteristic or hematochemical markers, gut morphometric indexes, and internal organs were all unaffected.

Oluokun [78] fed Anak red broiler chicks 5.6% BSFL along with SBM and observed that the weight gain was 2.02 kg, greater than that of SBM alone (1.47 kg) and comparable to that of FM (2.05 kg) supplementation at 4.5%. Additionally, it was indicated that BSFL meal might take the place of FM in broiler diets to greatly improve the nutritional value of SBM without having a negative impact on feed intake, weight gain, or FCR, which was shown to be 2.8 in SBM alone and 2.6 with BSFL supplemented close to FM (2.4). The Ardennaise chickens fed BSFL (8% inclusion) by Moula et al. [121] showed a significant difference in weekly weight gain but not in overall FCR, with the experimental group showing 4.01 and the control group showing 4.04. Elwert et al. [62] fed 6.6%, 5.4%, and 4.7% of BSFLM to broiler chickens. Compared to BM and FM, in the BSFLM group, FCR decreased by 6.6%. The daily weight gain is better with high inclusion of BSFLM. To bolster this, Ipema et al. [24] discovered that feeding live BSFL larvae at rates of 5% and 10% enhanced their health, foraging behavior, and overall activity while lowering their fear and immobility. The authors concluded that the BSSLM offers the poultry industry a viable substitute source of protein, oil, and animal welfare that can be utilized in a sustainable manner.

Moreover, it has been previously noted that *Salmonella enterica* and *Escherichia coli* O157:H7 disease-causing bacteria can be eliminated and rendered inactive by BSFL in the PL [30,31,127]. Lee et al. [23] reported a noteworthy observation regarding the prophylactic effects and stimulation of the non-specific immune system in Ross broilers when they were provided with a diet containing 1–3% BSFL. This dietary inclusion was found to enhance the immune system’s resistance to Salmonella, as evidenced by increased levels of CD4 cells, elevated serum lysogenic activity, and enhanced splenic lymphocytic proliferation in the chickens. Growth-wise, the chicks fed BSFL reached 1.3 kg in 30 days compared to 32 days for the control group. However, Muller et al. [127] hypothesized that because BSFL intestinal extracts have no effect on nematode eggs or coccidian oocysts, using BSFL as animal feed could increase the likelihood of parasite transmission, and larval washing is insufficient to eradicate parasites, indicating the necessity for additional hygiene measures.

### 4.2. BSFL in Layers

In order to replace 50% and 100% elimination of soybean cake, Maurer et al. [74] fed layer chickens a diet of 12% and 24% partially defatted BSFL. It is interesting to note that growth performance, egg output, feed intake, and yolk and shell weight all showed no discernible variations. No mortality or health problems were discovered among the treatments, and it was suggested that BSFL be used in place of SBM as a valuable layer’s diet. When Chu et al. [22] fed layer chickens with full-fatted BSFL for 1–42 days (beginning) with 3%, 6%, and 9% inclusion, the chickens gained 5.8% more weight and had a better FCR (2.12) than the control (2.23), which did not include any BSFL. Additionally, the BSFL supplemented layers showed improvements in the digestibility of crude protein and lipids, IgA secretions, and antioxidant chemicals. Significantly, controlled to BSFL, the mortality dropped from 8.5% to 5.8%. This study suggests that full-fat BSFLM inclusion can be a good source of protein for layer chickens throughout their transitional period. The egg-laying performance and cecal microbiota of layer chickens that were 168 days old were observed by Kawasaki et al. [37] after feeding them 10% full-fatted larvae and prepupae of BSF for 5 weeks. Interestingly, there was no discernible variation in the amount of feed consumed or the rate at which eggs were laid; yet, variations were observed in the weight and thickness of the eggshells, as well as in the cecal bacteria. Compared to the control feed (2.27), the FCR was better in the BSFL feed (2.01).

According to Patterson et al. [86], layer hens were fed BSFL oil (1.5%, 3%, and 4.5%) and meal (8%, 16%, and 24%) between the ages of 43 and 47 weeks and 51 and 55 weeks. With commercial hens, BSFL oil could easily replace soybean oil at inclusion levels of up to 4.5%. The oil treatments had no effect on daily feed consumption, body weight, egg production, FCR, or egg weight. BSFLM showed negative growth performance at the highest inclusion of 24%, while 8% and 16% BSFL meal showed similar to the control feed without any negative impact on performance.

Barragan-Fonseca et al. [3] observed a similar impact and reported that while BSFL can partially substitute traditional animal feed, a complete replacement results in subpar performance. Further research is needed on improved larval processing techniques and digestibility. In addition to expanding, commercial environments typically result in worsening animal welfare, which calls for improvement. Better leg health and broiler welfare may arise from providing an environment that encourages a wider variety of behaviors in chickens. The same effect was noticed by Barragan-Fonseca et al. [3], who stated that partial replacement of traditional animal feed is possible with BSFL, but complete replacement gives negative performance. The digestibility and improved methods to process larvae need more study. In addition to growth, animal welfare tends to be worsened under commercial settings and should be improved. Providing environmental conditions that facilitate a broader range of behaviors might increase chicken activity, resulting in superior leg health and broiler welfare.

## 5. Black Soldier Fly (BSF) in Circular Economy: Waste Management Model

Within a 12- to 14-day processing period, the BSF can consume a variety of organic wastes, including animal litter, food waste from homes and restaurants, food processing industry waste, municipal organic waste, brewery and distillery waste, and agricultural byproducts [28]. Its conversion efficiency is 15–25 kg waste/m^2^/day [128], 15–20% BCR [129], and 55–75% waste reduction. The essential amino acid profile of larval biomass is similar to that of fishmeal, and it comprises 40–45% protein and 28–35% fat, making it suitable for use in the animal feed industry [130]. According to Surendra et al. [131], the aquaculture feed sector may substitute 50% of fishmeal, 25–30% of poultry feed, and 36% of pet food formulations [132]. Lipid extraction efficiency of 30–35% was attained, and 100 L of biodiesel could be produced from 1000 kg of larvae [133]. It can be esterified for use as biodiesel [131]. Chitin, which can be recovered from larvae and cocoon shells at a rate of 5–7% of their dry weight, is utilized in cosmetics, pharmaceuticals, and biological applications [134]. Frass can be used on arable land, because it contains beneficial microorganisms, organic matter (50–60%), and nutritional content (NPK: 4:3:3) [129]. When compared to traditional techniques, BSF waste management lowers costs by 30–50%. Additionally, the larvae biomass has a market worth of USD 500–1000/ton, depending on the market, and it has the potential to produce value-added products and create jobs [29]. Additionally, BSF reduces greenhouse gas emissions (GHG) by 0.8–1.2 tons of CO_2_e/ton of waste [17], uses little water (1–2 L/kg waste), and uses less land (50–100 m^2^/ton of waste per day) [29].

## 6. Regulation in BSFL Biomass

In general, the utilization of manure-fed insect meal raises concerns regarding the bioaccumulation of contaminants [130]. The distribution of pollutants, especially heavy metals, is a significant concern during the conversion of organic waste into proteins and fats by insects. Research has shown that heavy metals tend to accumulate in different parts of the larval body during this process [128]. For example, cadmium (Cd) accumulates in the prepupae’s body, while zinc (Zn) and lead (Pb) concentrate in the discarded skin after molting. It is recommended to refrain from using prepupae as feed sourced from organic waste with high cadmium content. Nevertheless, Charlton et al. [135] conducted an investigation on the safety implications of BSFL exposed to various contaminants present in manure, such as veterinary drugs, pesticides, heavy metals, mycotoxins, dioxins, polychlorinated biphenyls, and polybrominated diphenyl ethers. Their study revealed that the levels of all these compounds in the larvae remained below the regulatory limits set by the European Union or Codex [130]. Wang et al. [130] observed a reduction in the bioaccumulation of heavy metals in the larvae aging, indicating the excretion of heavy metals from the larvae’s bodies as they grew. Consequently, the levels of heavy metals in the larvae met the standards for chicken feed, affirming the environmentally safe utilization of maggots in manure management.

The use of insect meal as food and feed was prohibited for a long time. Recently the use of BSFL as an animal feed has been recognized in the EU, Canada, the United States, and Australia under specific criteria ([136], EU regulation no 2015/2283. 1372/2021). The guidelines governing the raw materials used for insect production will influence the extent to which BSF larvae can be integrated into the food system. Currently, the European Union prohibits the use of insects that have been fed on materials such as manure or waste for feed applications [137]. However, the cultivation of BSFL on organic waste as a sustainable element in animal feed is receiving growing global interest. The extensive commercial implementation has been constrained by the lack of clearly established regulations, especially regarding the safety of larvae cultivated on different organic byproducts [136]. It is essential to acknowledge the capacity of BSFL to improve productivity and resource efficiency within food systems. Comprehending and complying with the regulations, which include aspects of waste management and feed safety, pose significant challenges for producers of BSFL. Understanding and adhering to the regulations, encompassing waste management and feed safety considerations, present notable challenges for BSFL producers. A clear understanding of the legal framework is essential for facilitating the industrial expansion of BSFL as animal feed [138].

## 7. Conclusions

For the sake of the poultry industry’s sustainability, the idea of a circular economy in chicken farms through the production of BSFL from PL is gaining traction. For successful continuous rearing, BSFL production requires a thorough understanding of the insect’s biology and practical applications. Better than SBM, BSFLM has high-quality nutrients similar to FM. PL is considered the ideal organic waste suitable for feeding to the BSFL, resulting in a satisfactory yield of high-quality animal feed and frasstilizer (organic fertilizer). The supplementation of BSFLM at a specific proportion enhances the performance of all growth stages in commercial poultry (layers and broilers). The conversion of PL into insect meal (IM) through this method is an environmentally friendly, sustainable, and promising technique for the poultry sector and other organic waste streams. However, it requires advancements in technology and practical aspects such as prolonging the lifespan of insects and egg storage (cryopreservation), enhancing the BCR of PL to BSFL and the FCR of BSFLM to chicken meat, and further research on the accumulation of contaminants in the larvae and its suitability as animal feed.

## Figures and Tables

**Table 2:** Food constituents of black soldier fly larvae (dry weight basis) grown in different organic waste.

Protein (%)	Fat (%)	Fiber (%)	Ash (%)	Water (%)	Energy	Organic Waste Fed	Reference
41.5	26.5	-	4.3	-	-	Food waste (pasta)	[74]
35	35.5	4.7 (chitin alone)	-	-	-	Veg waste	[22]
41.1–43.6	15–34.8	7	14.6–28.4%	-	5278.49 kcal/kg	-	[75,76,77]
	24.8	8.3	13.8	-	4750 Kcal/kg	Poultry litter	[78]
56 (larvae)	12.8	-	12.6	-	-	Chicken feed	[36]
52 (prepupae)	19.7	-	13.9	-	-	Chicken feed	[36]
42	35	-	-	57%	-	Chicken manure	[32]
41.1	30	22	9.3	-	-	CM	[79]
45.7	9.5	12.4	-	-	-	Spent sorghum and barley	[65]
50	35	-	-	-	-	Vegetable waste	[80]
39.9	37.1	-	9.6	59	-	Vegetable waste	[81]
43.1	38.6	-	2.7	61.9	-	Restaurant waste	[81]
41.2	33.6	-	10	61.3	-	Chicken feed	[81]
37–63	7–39	-	9–28	-	-	-	[3]
39.2	57.8	-	3.9	64.5	-	Bread	[64]
52.6	46.7	-	5.7	73	-	Fish	[64]
36.6	40.7	-	16.3	67	-	Food waste	[64]
42	29	-	-	-	-	-	[16]
47.6	11.8	-	-	-	-	Agricultural waste	[82]
43.2	28	-	16.6	-	-	Swine manure	[66]
42.1	34.8	7	14.6	-	-	Poultry manure	[66]
40	33	12	15	-	-	Mixed (plant and animal waste)	[83]
38–46	21–35	-	-	64.4–67.1	-	Food waste	[61]
42–44	31–35	-	11–15	-	-	Animal manure	[84]
37.8	41.7	-	-	-	-	Fruit waste	[85]
40.9	12.9	-	-	-	-	Horse manure	[85]
39.8	30.1	-	-	-	-	Organic mixed waste	[85]
46.3	11.9	7.4	10.8	-	-	-	[86]

**Table 3:** Biochemical constituents of poultry litter (dry weight basis).

pH	Protein (%)	OM (%)	Fat (%)	Carbohydrates (%)	Fiber (%)	Ash (%)	Ca (%)	C:N	Energy J/g	Reference
7.07		40.2% as TOC	-	-	-	-	-	15.7	-	[91]
-	25-32		2.3-5	-	11-15.7	22-35.6	-	-	-	[92]
--		58.6	-	-	-	-	0.4%	-	-	[93]
-	18.7	-	-	11	7	13	-	-	-	
-	-	62.5% VS	-	34.7 as OC	-	-	-	8.91	-	[94]
7.73	-	-		270 g/kg of carbon	-	-	-	5:1	-	[95]
-	~35	-	~10	~25	-	-	~1.5	3.15	-	[96]
6.10	34.5	-	-	21.12% as total C	-	-	0.16	3.83	6905	[97]
8.43	-	68.1%	-	39.5% as OC	-	31.9	-	11.4	-	[98]
-	-	-	-	27.8% as OC	-	-	1.4%	7.18	-	[99]

OM, organic matter; TOC, total organic carbon; VS, volatile solids; OC, organic carbon.

**Table 4:** Bioconversion of poultry litter into black soldier fly larvae, feed conversion ratio and waste reduction.

Chicken Manure (Substrate)	WR (%)	BCR (%)	FCR	Larva Age	Larva Developmental Time	Ref
CM	-	15.3	-	2nd DOL used,	8 days	[92]
CM + rice bran (15%)	-	20	-			
CM + Bacteria (BSFL associated bacteria)	53	10.44	5.55	6th DOL used,	14 days	[100]
CM + Chabazite	75	5.2	14.5	6th DOL used,	13 days	[101]
CM (batch fed)	74.1	10.5	7.1	3rd–5th DOL used, 150 mg CM/L/day	15 days	[102]
CM (Continuous fed)	80	12.8	8.2	220 mg CM/L/Day		
CM	22 (OM reduction)	-	-	6th DOL used,	9 days	[103]
CM	45	5.6	3.47	4th DOL used,	12 days	[20]
CM + *Bacillus subtilis* inoculation	40.5	11.5	-	6th DOL used,	13 days	[104]
CM	35.8	10.2				
CM	55	9.88	5.56 (dry wt basis) 1.28 (live wt basis)	6th DOL used,	18 days	[91]
CM (60%) and DM (40%)	52.1	7.86	6.63	6th DOL used,	20 days	
CM (dried and moistened)	37	3.4	2.3	<24h old larva used,	144 days	[61]
CM	48.4 (dry matter reduced)	-	-	6th DOL used,	13 daya	[105]
CM	>50	3.74	13.4	Eggs used	NA	[32]

CM, chicken manure; DM, dairy manure; WR, waste reduction; BCR, bioconversion rate; FCR, feed conversion ratio; DOL, day old larva; OM, organic matter; wt, weight.

**Table 5:** Chicken growth performance using BSFLM supplement.

Types of Chicken	BSFLM % and Others	Weight Gain/Day	FCR	Reference
Isa Brown (0–8 weeks)	25% + FM	9 g/day	2.78	[119]
Isa Brown (8–12 weeks)	25% + FM	10.2 g/day	1.45	[119]
Ross 308 (1–21 days)	50% + SBM +AA	19.7	1.23	[120]
Ross 308 (22–34 days)	50% + SBM +AA	101	1.29	[120]
Ross 308 (1–34 days)	50% + SBM +AA	62.5	1.26	[120]
Ross 308 (1–10 days)	10% + Maize and soy meal	24.5	1.16	[80]
Ross 308 (10–24 days)	10% + Maize and soy meal	67.3	1.47	[80]
Ross 308 (24–35 days)	10% + Maize and soy meal	95.6	1.83	[80]
Ross 308 (1–35 days)	10% + Maize and soy meal	62.5	1.60	[80]
Ardennaise chickens	8% BSFL	-	4.01	[121]
Anak red broiler (starter, 0–5 weeks)	5.6%	-	2.8	[78]
Anak red broiler (finisher, 5–10 weeks)	5.6%	-	2.4	[78]
Anak red broiler (whole, 0–10 weeks)	5.6%	6.7 g/day	2.6	[78]
Hy-line brown (layer) (1–42 days)	3	10.8 g/day	2.12	[22]
Hy-line brown (layer) (1–42 days)	6	10.5 g/day	2.14	[21]
Hy-line brown (layer) (1–42 days)	9	10.4 g/day	2.18	[22]
Julia laying hen (168 days used for 5 weeks exp)	10% larvae	35	2.20	[37]
Julia laying hen (168 days used for 5 weeks exp)	10% prepupae	38	2.01	[37]
Broiler (starter)	6.6	24.6	1.07	[62]
Broiler (starter)	5.4	23.4	1.08	[62]
Broiler (starter)	4.7	22.8	1.08	[62]
Broiler (finisher)	5	69.6	1.49	[62]
Broiler (finisher, 21–48 days)	50% replacement of soybean oil by BSFL fat	106	2.03	[122]
	100% replacement of soybean oil by BSFL fat	113	1.95	[122]
Broiler (1–35 days)	1.5% BSFL oil	49.5	1.65	[123]
	1.5% BSFL oil	49.8	1.60	[123]
Layer hen 43–47 weeks	4.5% BSFL oil	-	1.89	[86]
Layer hen 51–55 weeks	16% BSFL		1.85	[86]

BSFLM, black soldier fly larvae meal; FM, fishmeal; SBM, soybean meal; AA, amino acid; FCR, feed conversion ratio. Note: the substrates utilized for the rearing of black soldier fly larvae (BSFL) consist of wood waste, distillery byproducts, fruit refuse, and poultry feed.

## Abbreviations

PL: poultry litter; BSF, black soldier fly; BSFL, black soldier fly larvae; BSFLM, black soldier fly larvae meal; IM; insect meal; BCR, bioconversion rate; FCR, feed conversion ratio; WR, waste reduction; OW, organic waste; WRI, waste reduction index; SR, survival rate; GR, growth rate; EFP, efficiency of frass production; FM, fishmeal; SBM, soybean meal; PM, pig manure, DM, dairy manure; CM, chicken manure.
